# Supplementary material for: Assessment of the Most Impactful Combination of Factors Associated with Nocturia and to Define Nocturnal Polyuria by Multivariate Modelling
Source: J Clin Med. 2020 Jul 16;9(7):2262. doi: 10.3390/jcm9072262 (PMC7408683; doi:10.3390/jcm9072262)
Supplement: Supplementary file 1 [file jcm-09-02262-s001.pdf]

Supplementary Materials

Table S1. Significance level of univariate analysis of variables on a per patient basis.

Variable	All	000085	CS40	CS41
NOCVD	***	***	***	***
NI	***	***	***	***
NI_gt_1.5	***	***	***	***
NI_gt_1	***	***	***	***
SD	***	***	***	***
NPI	***	**	*	**
NUPw	***	***		**
maxVV	***		***	***
NUPh	***	***		
NPI_bin	***			**
NPIh	***			
ASEX	***			
NUPw_gt_10	***	**		
NUPh_gte_54	**	***		
NUPh_gt_90	**	***		
NUPh_gte_78	**	***		
ARACE	*		-	
VOL24HR		***		
AGE				
BMIBL		-		
Intake_N			*	
Intake_1htb				

0 - 0.001 `***′; 0.001 - 0.01 `**′, 0.01 - 0.05 `*′, 0.05 - 0.1 `-′, 0.1 - 1.0 `′ (no symbol); ASEX: gender at birth; ARACE: race; Bin: binary; BMI: body mass index; Gt: greater than; Gte: greater than or equal to; Intake N: intake night; Intake 1htb: intake 1 hour before going to bed; maxVV: maximum voided volume; NOCVD: nocturnal voids; NI: nocturnal Index; NUPh: nocturnal urine production per hour; NUPw: nocturnal urine production per weight; NPI: nocturnal polyuria index; SD: Sleep duration;VOL24HR: total voided volume over 24 h.
